# Benefits of neoadjuvant chemotherapy: is the prognosis of ypN0 patients after neoadjuvant chemotherapy comparable to that of pN0 patients undergoing surgery alone?

**DOI:** 10.1007/s10388-025-01132-9

**Published:** 2025-05-20

**Authors:** Osamu Shiraishi, Koji Tanaka, Tomoki Makino, Takahito Sugase, Takashi Kanemura, Atsushi Takeno, Keijiro Sugimura, Masaaki Motoori, Yutaka Kimura, Motohiro Hirao, Kazumasa Fujitani, Hiroshi Miyata, Masahiko Yano, Makoto Yamasaki, Yuichiro Doki, Takushi Yasuda

**Affiliations:** 1 Department of Surgery, Kindai University Faculty of Medicine, 377-2 Ohnohigashi, Osaka-Sayama, Osaka, 589-8511 Japan; 2 Department of Gastroenterological Surgery, Graduate School of Medicine, Osaka University, Suita, Osaka Japan; 3 Department of Gastroenterological Surgery, Osaka International Cancer Institute, Osaka, Japan; 4 Department of Surgery, National Hospital Organization, Osaka National Hospital, Osaka, Japan; 5 Department of Surgery, Kansai Rosai Hospital, Hyogo, Japan; 6 Department of Surgery, Osaka General Medical Center, Osaka, Japan; 7 Department of Surgery, Kindai University Nara Hospital, Nara, Japan; 8 Kyowakai Hospital, Osaka, Japan; 9 Department of Surgery, Kansai Medical University, Hirakata, Osaka Japan

**Keywords:** Esophageal cancer, Neoadjuvant chemotherapy, YpN0

## Abstract

**Background:**

Preoperative treatment has become widely recognized for improving survival in patients with esophageal cancer. The present study aimed to compare the prognosis between patients with pathological node-negative status treated with surgery alone (SA-pN0) and those who were clinically node-positive but converted to ypN0 following neoadjuvant chemotherapy (NAC-ypN0) in cases of advanced thoracic esophageal squamous cell carcinoma (ESCC).

**Methods:**

This retrospective analysis used a multicenter database of 4849 consecutive patients who underwent treatment for esophageal cancer. Patients with clinical T2 or more advanced ESCC who underwent standard subtotal esophagectomy between 1990 and 2017 were included. The NAC-ypN0 group was compared with the SA-pN0 group in terms of patient characteristics, recurrence patterns, and survival outcomes using propensity score-matched analysis.

**Results:**

In total, 109 patients were classified as NAC-ypN0 and 137 as SA-pN0. Propensity score matching resulted in the selection of 87 patients per group. Compared with the SA-pN0 group, the NAC-ypN0 group had a significantly more advanced clinical TNM stage and underwent significantly more three-field lymphadenectomies. Pathological findings showed downstaging of the pT stage in the NAC-ypN0 group, resulting in an equivalent distribution between the two groups. Additionally, the NAC-ypN0 group had significantly lower rates of lymphatic invasion (33% vs. 56%) and venous invasion (21% vs. 52%). Recurrence rates (21% vs. 22%) and survival outcomes (5-year overall survival: 83.9% vs. 76.1%, *P* = 0.110) were comparable between the two groups.

**Conclusions:**

The NAC-ypN0 group demonstrated reduced lymphovascular invasion and showed a prognosis comparable to that of the SA-pN0 group.

## Introduction

In the treatment of stage II/III thoracic esophageal cancer, preoperative therapy followed by radical resection has been established as the standard approach, with evidence demonstrating an improved prognosis. In Western countries, neoadjuvant chemoradiation (NACRT) followed by esophagectomy, as shown in the CROSS trials, is the standard treatment and yields better outcomes than surgery alone [1, 2]. More recently, the ESOPEC trial showed that perioperative chemotherapy with fluorouracil, leucovorin, oxaliplatin, and docetaxel plus surgery resulted in improved survival in patients with resectable esophageal adenocarcinoma compared with preoperative chemoradiotherapy plus surgery [3]. In Japan, the JCOG9904 trial demonstrated that neoadjuvant chemotherapy (NAC) using cisplatin and 5-fluorouracil (CF) followed by esophagectomy improved the prognosis compared with adjuvant CF after surgery [4], establishing NAC as the standard treatment. Furthermore, the JCOG1104 trial—a three-arm comparison of NAC with docetaxel plus CF (DCF), NACRT, and NAC-CF—showed that NAC-DCF, but not NACRT, significantly improved the prognosis, leading to the recognition of DCF-NAC as the new standard treatment [5].

Both NACRT and NAC as preoperative treatments lead to tumor regression in the primary tumor and lymph node metastases, thereby improving the prognosis after surgery. The antitumor effect on the primary lesion has a prognostic impact that correlates with the degree of response as evaluated by systems such as the Mandard tumor regression grade or the Japan Esophageal Society classification [6, 7]. Similarly, the effect of preoperative treatment on lymph node metastases is also an important prognostic factor. It has been reported that the status of lymph node metastasis is an independent prognostic indicator, and the disappearance of lymph node metastases clearly improves the prognosis [8–11]. Moreover, by considering both histomorphological tumor regression and lymph node status, we can more accurately predict patient outcomes [12–15]. In this context, a simple yet intriguing question arises: Do patients with natural pN0 status who undergo upfront surgery have the same disease characteristics and prognosis as those who initially had clinically positive lymph nodes but converted to ypN0 after NAC? To date, no studies have addressed this question in patients who received NAC for esophageal squamous cell carcinoma (ESCC). Therefore, we have focused our attention on this very issue.

We are collaborating with Osaka University, Kindai University, and related institutions to build a large database on esophageal cancer treatment and to conduct research aimed at answering various clinical questions. This database comprises patients who were treated using a uniform therapeutic strategy and consistent surgical techniques, offering the advantage of minimal institutional treatment bias. Leveraging this robust resource, the purpose of the present study was to compare the prognosis of the natural pN0 group (patients treated with upfront surgery) with that of the ypN0 group (patients whose cN1 status converted to negative following NAC for advanced esophageal cancer).

## Patients and methods

### Patient eligibility criteria

This research was conducted as a retrospective analysis using a comprehensive database. Data were collected from medical records in an esophageal cancer database comprising 4849 consecutive patients who underwent esophagectomy for esophageal cancer between 1965 and 2017 at Osaka University Hospital, Osaka International Cancer Institute, Kindai University Hospital, Kansai Rosai Hospital, National Osaka Hospital, or Osaka General Medical Center. Patients with advanced thoracic ESCC—defined as clinical T2 or higher—who underwent a typical subtotal esophagectomy via a right thoracic approach with two- or three-field lymphadenectomy after January 1990 were eligible for inclusion. Patients were excluded if they met any of the following criteria: received preoperative radiation treatment, had residual tumors or distant metastases, had a history of advanced cancer other than esophageal cancer within the past 5 years, died of treatment-related causes, underwent a second-stage operation, had pathological lymph node metastasis, had clinical N0 status with NAC, had clinical T1 or T4 stage disease, or had missing data.

Patients pathologically classified as N0 were divided into two groups: natural pN0, who underwent upfront surgery (SA-pN0), and ypN0, whose cN1–3 status converted to negative following NAC (NAC-ypN0). Both groups were evaluated in terms of clinical status, histopathological characteristics, recurrence rate, overall survival (OS), recurrence-free survival (RFS), and cancer-specific survival (CSS).

The diagnosis of clinical lymph node metastases was based on 5-mm-slice computed tomography scans and recorded in the database at each participating institution. Clinical and pathological stages were classified according to the Union for International Cancer Control TNM Classification of Malignant Tumors, 7 th edition. The study protocol was approved by the review board of each institution prior to patient enrollment in accordance with the principles of the Declaration of Helsinki (Approval Number 29–214). An opt-out method for informed consent was used, with study details presented on each institution’s website.

### Propensity score matching

This study compared downstaged ypN0 cases from cN1 following NAC with natural pN0 cases treated with surgery alone. While differences in clinical TNM stages and the field of lymphadenectomy were anticipated, the pathological T stage was adjusted to achieve comparability using propensity score-matched analysis. To account for additional factors, propensity score matching was performed using a logistic regression model incorporating the following covariates: sex, age, tumor location, and pathological T stage.

### Statistical analysis

Continuous variables are presented as means and medians. The *t* test was used to compare parametric variables, and the Mann–Whitney *U* test was used for nonparametric variables. Categorical data were compared using Fisher’s exact test or Pearson’s Chi-square test. OS, RFS, and CSS were estimated using the Kaplan–Meier method. OS was defined as the time from surgery to death of any cause, RFS as the time from surgery to recurrence or death, and CSS as the time from surgery to cancer-related death. A *P* value of < 0.05 was considered statistically significant. All statistical analyses were performed using JMP data analysis software, version 18.0.0 (SAS Institute Inc., Cary, NC, USA). The propensity score matching analyses were performed with EZR (Jichi Medical University, Tochigi, Japan), which is a graphical user interface for modified version of R commander (The R Foundation for Statistical Computing, Vienna, Austria).

## Results

### Patients

**Fig. 1:**
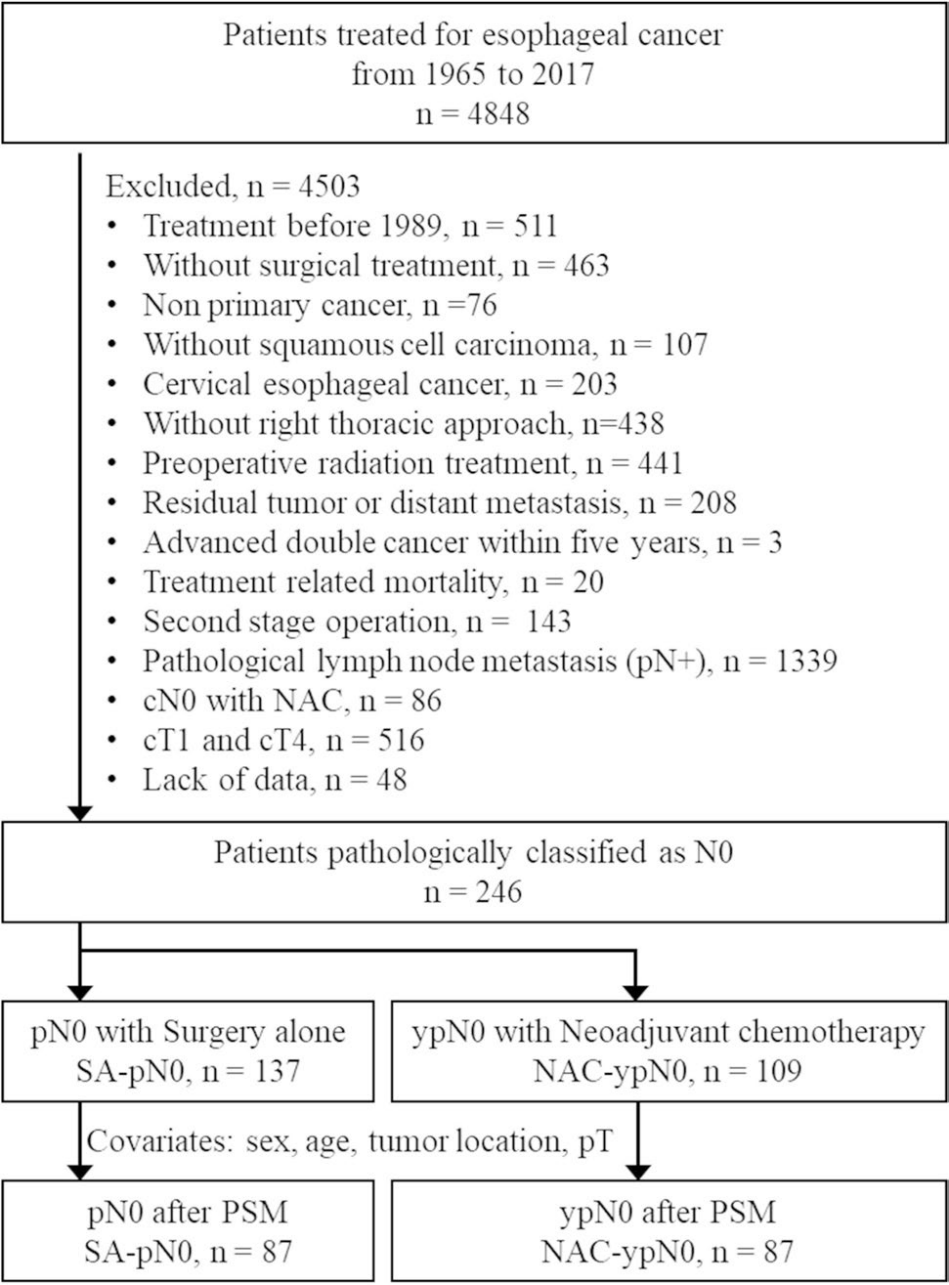
CONSORT flow diagram

A total of 246 patients met the eligibility criteria, comprising 109 patients in the NAC-ypN0 group and 137 patients in the SA-pN0 group. Propensity score matching analysis, using age, sex, tumor location and pathological T stage as covariates, resulted in the selection of 87 patients in each group. (Fig. 1). Patients’ characteristics are summarized in Table 1. Compared with the SA-pN0 group, the NAC-ypN0 group, converted from the cN1–3 group, exhibited a significantly more advanced clinical TNM stage. Additionally, patients in the NAC-ypN0 group tended to be younger. Regarding treatment regimens, CF accounted for 11%, adriamycin + CF for 62%, and DCF for 14% of the NAC treatments. The NAC-ypN0 group also underwent significantly more three-field lymphadenectomy procedures than the SA-pN0 group, and the number of lymph node dissections tended to be higher. Following propensity score matching, age and sex distributions were well-balanced between the two groups.

**Table 1:** Patient characteristics

	NAC-ypN0	SA-pN0	*P* value	SMD	NAC-ypN0	SA-pN0	*P* value	SMD
	*N* = 109 (%)	*N* = 137 (%)			*N* = 87 (%)	*N* = 87 (%)		
Age (SD)	63.7 ± 8.0	65.5 ± 8.3	0.086	0.222	64.3 ± 6.9	64.0 ± 7.0	0.776	0.043
Sex			0.272	0.140			1.000	< 0.001
Male	82 (75)	111 (81)			68 (78)	68 (78)		
Female	27 (25)	26 (19)			19 (22)	19 (22)		
Location			0.341	0.188			0.972	0.049
Upper	10 (9)	15 (11)			7 (8)	7 (8)		
Middle	55 (50)	79 (58)			45 (52)	47 (54)		
Lower	44 (40)	43 (31)			35 (40)	33 (38)		
cT stage			< 0.001	0.823			< 0.001	0.771
cT2	28 (26)	87 (64)			22 (25)	53 (61)		
cT3	81 (74)	50 (36)			65 (75)	34 (39)		
cN stage			< 0.001	2.943			< 0.001	3.374
cN0	1 (1)	113 (82)			0	74 (85)		
cN1–3	108 (99)	24 (18)			87 (100)	13 (15)		
cM stage			0.046	0.277			0.121	0.310
cM0	103 (95)	136 (99)			83 (95)	87 (100)		
cM1LYM (only SCL)	6 (5)	1 (1)			4 (5)	0		
cStage			< 0.001	3.092			< 0.001	3.170
IIA	0	113 (82)			0	74 (85)		
IIB	23 (21)	8 (6)			19 (22)	3 (3)		
III	80 (73)	15 (11)			64 (74)	10 (11)		
IV	6 (6)	1 (7)			4 (5)	0		
NAC regimen								
CF	12 (11)				9 (10)			
ACF	68 (62)				69 (68)			
DCF	15 (14)				12 (14)			
Unknown	14 (13)				7 (8)			
Esophagectomy			0.137	0.217			0.246	0.267
McKowen	108 (99)	131 (96)			87 (100)	84 (97)		
Ivor Lewis	1 (1)	6 (4)			0	3 (3)		
Field of lymphadenectomy			< 0.001	0.606			< 0.001	0.602
2-FL	33 (30)	81 (59)			28 (32)	53 (61)		
3-FL	76 (70)	56 (41)			59 (78)	34 (39)		
Number of dissected LN (SD)	72 ± 28	64.7 ± 33	0.063	0.242	71.9 ± 27	67.9 ± 36	0.395	0.129

*NAC* neoadjuvant chemotherapy, *SA* surgery alone, *SD* standardized deviation, *SCL* supra clavicular lymph node, *LN* lymph node, *SMD* standardized mean difference, *CF* cisplatin + fluorouracil, *ACF* adriamycin + CF, *DCF* docetaxel + CF

The histopathological findings are presented in Table 2. The pathological T stage in the NAC-ypN0 group was downstaged, becoming comparable to that in the SA-pN0 group. Reflecting this downstaging of the primary tumor, both lymphatic and vascular invasion were significantly lower in the NAC-ypN0 group than in the SA-pN0 group, with lymphatic invasion observed in 30% vs. 56% and venous invasion in 18% vs. 40%, respectively. After propensity score matching, ypT0 cases were excluded, and the pathological T stage became nearly equivalent between the groups. Both lymphatic and vascular invasion remained significantly lower in the NAC-ypN0 group.

**Table 2:** Histopathological findings

	NAC-ypN0	SA-pN0	*P* value	SMD	NAC-ypN0	SA-pN0	*P* value	SMD
	*N* = 109 (%)	*N* = 137 (%)			*N* = 87 (%)	*N* = 87 (%)		
pT stage			< 0.001	0.616			0.960	0.054
pT0	14 (13)	0			0	0		
pT1	23 (21)	44 (32)			22 (25)	23 (36)		
pT2	23 (21)	32 (23)			22 (25)	20 (23)		
pT3	49 (45)	60 (44)			43 (49)	44 (51)		
pT4	0	1 (1)			0	0		
pT grade								
3	14 (13)				0			
2	28 (26)				25 (29)			
1b	19 (17)				18 (21)			
1a	35 (32)				32 (37)			
0	4 (4)				4 (5)			
Unknown	9 (8)				0			
Lymphatic invasion			< 0.001	0.565			0.002	0.514
ly+	33 (30)	77 (56)			29 (33)	49 (56)		
ly−	76 (70)	59 (43)			58 (67)	37 (43)		
Unknown	0	1 (1)			0	1 (1)		
Venous invasion			< 0.001	0.515			< 0.001	0.715
v+	20 (18)	55 (40)			18 (21)	45 (52)		
v−	89 (82)	81 (59)			69 (79)	41 (47)		
Unknown	0	1 (1)			0	1 (1)		

*NAC* neoadjuvant chemotherapy, *SA* surgery alone, *SMD* standardized mean difference

### Recurrence and prognosis

**Fig. 2:**
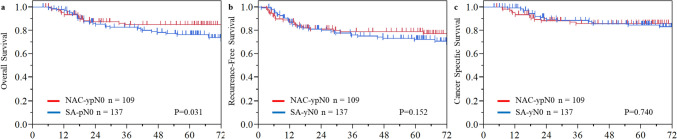
Kaplan–Meier estimates of **a** overall survival, **b** recurrence-free survival, and **c** cancer-specific survival in the NAC-ypN0 and the SA-pN0 groups in the entire cohort

**Fig. 3:**
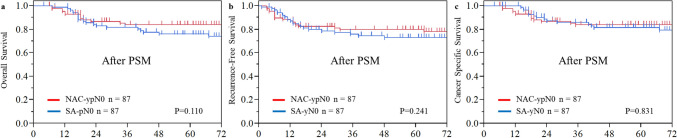
Kaplan–Meier estimates of **a** overall survival, **b** recurrence-free survival, and **c** cancer-specific survival in the NAC-ypN0 and the SA-pN0 groups after propensity score matching

**Fig. 4:**
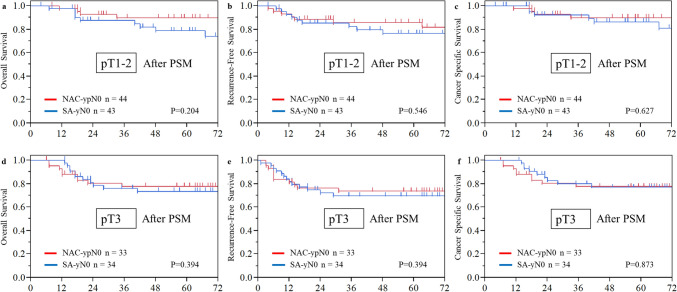
Kaplan–Meier estimates of **a**, **d** overall survival, **b**, **e** recurrence-free survival, and **c**, **f** cancer-specific survival in the NAC-ypN0 and SA-pN0 groups, evaluated in pT0–2 and pT3–4 cases for subgroup analysis after propensity score matching

The median follow-up period for censored patients was 67.7 months (interquartile range 46.5–91 months). Details of recurrence are presented in Table 3. The recurrence rate was similar between the two groups: 20% in the NAC-ypN0 group and 21% in the SA-pN0 group. Although blood-borne metastases tended to be more frequent in the NAC-ypN0 group, no statistically significant differences were observed in recurrence patterns. After propensity score matching, the recurrence rate was similar between the two groups, consistent with the findings in the overall cohort. The 5-year OS rate was significantly higher in the NAC-ypN0 group than in the SA-pN0 group (85.0% vs. 76.4%, *P* = 0.031), while the RFS rate was 78.9% versus 72.4% (*P* = 0.152), indicating a trend toward better survival in the NAC-ypN0 group. However, CSS—excluding deaths of other causes—was comparable between the groups, at 85.9% and 84.6% (*P* = 0.740) (Fig. 2). After propensity score matching, the 5-year OS rates were 83.9% for the NAC-ypN0 group and 76.1% for the SA-pN0 group (*P* = 0.110). The RFS rates were 79.8% and 73.1% (*P* = 0.241), while the CSS rates were 83.9% and 81.6% (*P* = 0.831), respectively. None of these survival outcomes demonstrated a statistically significant difference between the groups following adjustment (Fig. 3). Subsequently, OS, RFS, and CSS were assessed according to pathological T stage following propensity score matching. In both the pT1–2 and pT3 subgroups, survival outcomes were comparable between the NAC-ypN0 and SA-pN0 groups (Fig. 4).

**Table 3:** Long-term outcomes

	NAC-ypN0	SA-pN0	*P* value	SMD	NAC-ypN0	SA-pN0	*P* value	SMD
	*N* = 109 (%)	*N* = 137 (%)			*N* = 87 (%)	*N* = 87 (%)		
With recurrence	22 (20)	29 (21)	0.849	0.024	18 (21)	19 (22)	1.000	0.028
Recurrence pattern								
Lymph node recurrence	11 (10)	16 (12)	0.692	0.051	8 (9)	9 (10)	1.000	0.039
Blood-borne metastasis	12 (11)	7 (5)	0.097	0.218	11 (13)	5 (6)	0.188	0.240
Lung	5	2			5	1		
Liver	4	3			4	2		
Bone	1	2			1	1		
Brain	3	0			2	0		
Dissemination	1 (1)	3 (2)	0.632	0.103	1 (1)	2 (2)	1.000	0.088
Local recurrence	4 (4)	5 (4)	0.993	0.001	3 (3)	5 (6)	0.720	0.110

*NAC* neoadjuvant chemotherapy, *SA* surgery alone, *SMD* standardized mean difference

## Discussion

Using a multi-institutional database, we compared downstaged ypN0 cases—originally cN1—after NAC with natural pN0 cases treated with surgery alone among patients with advanced stage II/III thoracic ESCC who underwent radical esophagectomy. Even among patients with advanced cT factors and clinically positive lymph node status prior to treatment, the group in which NAC effectively converted lymph node metastases to negative (ypN0) showed T factor downstaging and a lower incidence of lymphovascular invasion. As a result, recurrence and survival rates in the ypN0 group were comparable to that in the natural pN0 group in the SA group.

In the present study, investigating cases of advanced ESCC, patients in the downstaged NAC-ypN0 group achieved a recurrence rate equivalent to that in the natural SA-pN0 group. The fact that lymphovascular invasion—a known prognostic marker—was lower in the NAC-ypN0 group than in the SA-pN0 group supports the idea that NAC contributes to an improved prognosis [16]. In reviewing past studies, some discrepancies emerge, such as differences in histological type (e.g., adenocarcinoma) or the use of NACRT. Table 4 summarizes five retrospective studies that focused on downstaged ypN0 cases. Leers et al. and Depypere et al. compared SA-pN0 with NACRT-ypN0 in patients with esophageal adenocarcinoma and reported that the 5-year OS rate for SA-pN0 ranged from 85.0 to 65.9%, which was significantly higher than that for NACRT-ypN0 (49.0–38.6%) [17, 18]. Noble et al. also compared SA-pN0 with NAC-ypN0 in patients with adenocarcinoma and reported a 5-year DFS rate of 80% for pN0 and 64% for ypN0 [19]. Although the prognosis for SA-pN0 was significantly better, the NAC-ypN0 group still demonstrated a relatively favorable outcome in that study. In these reports, approximately half of the patients in the SA group were classified as T1 N0, which naturally led to a good prognosis and, consequently, made survival differences between the two groups inevitable. We believe that the present study is meaningful in that it utilized propensity score matching-adjusted data to present survival rates for each pT stage and demonstrated their equivalence. Three additional reports focused on patients treated with NACRT, comparing cN0ypN0 with cN + ypN0 cases based on either preoperative diagnosis or pathological findings. Depypere et al. reported that the 5-year OS rate for adenocarcinoma was 58.2% in cN0ypN0 cases and 38.6% in cN + ypN0 cases [18]. Similarly, Zanoni et al. reported that the 5-year OS rate for adenocarcinoma/squamous cell carcinoma was 79% in cN0ypN0 cases and 41% in cN + ypN0 cases [14]. They concluded that ypN0 with prior lymph node metastasis was associated with a poorer prognosis. However, Hsu et al. reported no significant difference in 5-year OS between cN0ypN0 (42.1%) and cN + ypN0 (52.8%) in squamous cell carcinoma [20]. These findings suggest that the prognostic benefit of preoperative treatment may be greater in squamous cell carcinoma than in adenocarcinoma as also indicated by the results of the CROSS trial [1]. The reason our study found comparable recurrence rates and survival rates between NAC-ypN0 and SA-pN0 is likely due to several factors: the emphasis on lymph node metastasis as a key prognostic indicator and surrogate for systemic disease; the exclusive inclusion of patients with advanced SCC; and the use of NAC, which is expected to have a stronger systemic effect than CRT, which primarily provides local control.

**Table 4:** Characteristics of study population

Author	Year	Histology	Treatment	Group (*n*)	cN0/cN+	cT1/2/3/4	pT0/1/2/3/4	LVI		NLND	5y-OS	5y-DFS
								Ly+	V+			
Leers [17]	2009	AC	SA	pN0(75)	NS	NS	0/51/11/13/0	23%		45	85%	NS
			NCRT/NAC	ypN0(25)	NS	NS	10/5/4/6/0	24%		32	49%	NS
Noble [19]	2013	AC	SA	pN0(40)	27/13	13/17/10/0	2/22/5/10/0	5%	17.5%	NS	NS	80%
			NAC	ypN0(73)	13/60	0/9/61/3	11/20/20/24/1	8.2%	13.7%	NS	NS	64%
Depypere [18]	2021	AC	SA	pN0(45)	cN+	8/17/20/0	0/23/6/16/0	NS		24	65.9%	63.3%
			SA	pN+(143)	cN+	3/30/108/2	0/5/19/113/6	NS		32	24.5%	21.7%
		AC	NCRT	ypN0/LNR-(55)	cN+	0/11/37/7	18/7/14/16/0	NS		23	58.2%	54.5%
			NCRT	ypN0/LNR+(44)	cN+	1/10/33/0	14/8/6/16/0	NS		26	38.6%	31.8%
			NCRT	ypN+(95)	cN+	0/8/80/7	7/15/11/60/0	NS		28	28.4%	21.7%
Zanoni [14]	2016	AC/SCC	NCRT	ypN0(28)	cN0	NS	NS	NS		17	79%	79%
			NCRT	ypN0(37)	cN+	NS	NS	NS		20	41%	45%
			NCRT	ypN+(18)	cN+	NS	NS	NS		22.5	0%	0%
Hsu [20]	2021	SCC	NCRT	ypN0/LNR-(73)	15/58	2/15/52/4	35/8/11/17/2	12.3%		NS	42.1%	38.1%
			NCRT	ypN0/LNR+(25)	4/21	1/4/19/1	19/3/2/1/0	0.0%		NS	52.8%	45.3%
			NCRT	ypN+(38)	2/36	2/7/31/1	6/6/7/17/2	39.5%		NS	8%	5%
Present	2025	SCC	SA	pN0(137)	113/24	0/87/50/0	0/44/32/30/1	56%	40%	64.7	76.4%	72.4%
			NAC	ypN0(109)	cN+	0/25/81/0	14/23/23/49	30%	18%	72	85%	78.9%

*AC* adenocarcinoma, *SCC *squamous cell carcinoma, *SA* surgery alone, *NCRT* neoadjuvant chemoradiation, *NAC* neoadjuvant chemotherapy, *NS* not specified, *LVI* lymphovascular invasion, *NLND* number of lymph node dissection, *OS* overall survival, *DFS* disease-free survival, *LNR* lymph node response

In the present study, although there was no significant difference in the recurrence rate or 5-year CSS between the two groups, a significant difference was observed in 5-year OS in all cohort data. Because this was a multi-institutional database study, detailed treatment selection bias could not be fully assessed. Given that upfront surgery was selected despite advanced esophageal cancer, it can be inferred that the SA-pN0 group included a substantial number of patients who were unable to receive NAC because of frailty. As a result, there were more deaths of other causes in the SA-pN0 group than in the NAC-ypN0 group, which likely accounts for the observed difference in OS. In fact, the present study demonstrated that the recurrence rate in the NAC-ypN0 group improved to a level comparable to that of the natural pN0 group. Moreover, propensity score matching confirmed that the survival rates were comparable between the groups across all endpoints. These findings provide valuable evidence supporting the role of NAC in improving the survival prognosis.

Naturally, the present study has some limitations. As mentioned earlier, it was a retrospective study using a large database involving the participation of multiple institutions. Patients with esophageal cancer often have backgrounds that contribute to frailty—such as a history of heavy drinking, smoking, advanced age, or difficulty with oral intake—so treatment selection bias was inevitably present. In particular, long-term survival outcomes, such as OS and RFS, are influenced not only by tumor-related factors but also by mortality from other diseases. The database did not contain comprehensive or precise data regarding patients’ frailty and comorbidities. Although strict adjustment for these factors was not feasible, propensity score matching incorporating age, sex, tumor location, and pathological T stage as covariates demonstrated generally comparable prognoses between the groups. That said, the esophageal cancer database used in the present study—comprising Osaka University, Kindai University, and their affiliated institutions—was established by centers with extensive experience in esophageal cancer treatment. This is an advantage because the study is based on a database with minimal institutional bias, where patients were treated using consistent therapeutic strategies and surgical techniques.

## Conclusions

Is the prognosis of ypN0 patients after neoadjuvant chemotherapy comparable to that of pN0 patients undergoing surgery alone? To investigate this, we compared downstaged ypN0 cases originating from cN1 after neoadjuvant chemotherapy with natural pN0 cases treated with surgery alone among patients with advanced thoracic esophageal squamous cell carcinoma who underwent radical esophagectomy. The group in which neoadjuvant chemotherapy effectively converted cN1 to ypN0 exhibited T factor downstaging and a lower incidence of lymphovascular invasion. Consequently, the recurrence prognosis of the ypN0 group was comparable to that of the natural pN0 group in the surgery alone cohort.

## Data Availability

The data that support the findings of this study are available from the corresponding author, O.S, upon reasonable request.
